# Therapeutic Effects of Xianlu Oral Solution on Rats with Oligoasthenozoospermia through Alleviating Apoptosis and Oxidative Stress

**DOI:** 10.1155/2022/1269530

**Published:** 2022-06-18

**Authors:** Zi-Run Jin, Ya-Lei Cao, Zhi-Chao Luo, Qian-Cheng Zhao, Yu Xi, Jia-Ming Weng, Zhe Zhang, Hui Jiang

**Affiliations:** ^1^Department of Urology, Peking University Third Hospital, Beijing, China; ^2^Department of Reproductive Medicine Center, Peking University Third Hospital, Beijing, China

## Abstract

Idiopathic oligoasthenozoospermia (iOAZS) is one of the major causes of male infertility, and the ideal therapies for iOAZS have not been established yet. Traditional Chinese medicine (TCM), including Xianlu oral solution (XL), has been widely used as an adjunct treatment for male infertility in the clinic. However, the underlying mechanisms of XL treatment on iOAZS are still not known. Here, we found that XL treatment has therapeutic effects on ornidazole (ORN)-induced OAZS model rats through the amelioration of testis tissues spermatogenesis and the improvement of sperm concentration and motility. Moreover, XL treatment ameliorated the serum hormone levels, mitochondrial membrane potential, apoptosis status, and oxidative stress status in the testis tissues of iOAZS model rats. These findings identify a potential mechanism underlying the therapeutic effects of Xianlu oral solution on iOAZS, and Xianlu oral solution may be used as a traditional Chinese medicine (TCM) therapy for male infertility caused by iOAZS in clinical practice.

## 1. Introduction

Male infertility is an emerging global public health issue. Approximately 7% of the male population is diagnosed with some type of infertility, such as asthenozoospermia, oligozoospermia, teratozoospermia, some combination of them, or azoospermia [1, 2]. Approximately 19% and 63% of these men with infertility were categorized as men having asthenozoospermia (AZS), combined with oligozoospermia (OZS), and/or teratozoospermia, respectively [3]. A multitude of causes can lead to asthenozoospermia (AZS) or oligozoospermia (OZS) including gene abnormality [4–9], unhealthy lifestyle, prolonged duration of sexual abstinence, infection, abnormal immunity, and urogenital diseases [10–12]. However, no clear causes have been diagnosed in some cases using routine clinical examinations, and these cases have been categorized as idiopathic AZS or OZS (iAZS or iOZS). Some therapies for AZS or OZS have been established such as treating infection or varicocele by antibiotics or surgery, changing lifestyle, avoiding toxic environmental exposures, and maintaining regular intercourse and ejaculation [13–16]. Additionally, some severe cases of AZS or OZS caused by genetic factors benefit from the application of intracytoplasmic sperm injection (ICSI) [17]. However, ideal therapies for iAZS or iOZS have not been established.

Traditional Chinese medicine (TCM) has been widely used as an adjunctive treatment for many kinds of infertility. It has been reported that upregulation of CatSper1 (cation channel of sperm) by Sheng-Jing-San, a TCM recipe treatment, improves the sperm motility of AZS rats and that oral administration of Wuzi Yanzong (WZYZ) formula can restore the destroyed testicular structure of oligoasthenozoospermic model rats [18, 19]. *Cistanche tubulosa* (CT), echinacoside (ECH), and phenylethanol glycosides from *C. tubulosa* (CPhGs) could also attenuate poor sperm quality and testicular toxicity through upregulation of steroidogenic enzymes via the CYP450-3*β*-HSD pathway in Leydig cells of bisphenol A- or hydrocortisone-induced animal models [20, 21]. Additionally, studies have found that new Wenshen Shengjing decoction (WSSJD) treatment could repair cyclosporine-induced testicular damage in mice by increasing testosterone levels in the testes and decreasing the apoptosis of spermatogenic cells; *Cuscuta chinensis* Lam. and *Lycium barbarum* L. treatment could regulate the expression of Bcl-2, BAD, and BAX, thus reducing cell apoptosis and improving sperm counts and the viability of *Tripterygium wilfordii* Hook. (GTW) polyglycoside-treated rats [22, 23].

Xianlu oral solution (XL), consisting of several traditional Chinese medicines, has been used clinically in China for the treatment of men with infertility with decreased sperm count and motility induced by kidney-yin deficiency. However, the effect and underlying mechanisms of Xianlu oral solution for the treatment of iOAZS remain unknown. In the present study, we first investigated whether XL treatment exerts its action on iOAZS model rats. Then, we investigated the possible underlying mechanisms for its treatment of iOAZS.

## 2. Materials and Methods

### 2.1. Animals

Sexually mature male Sprague-Dawley rats, weighing 180–200 g at the beginning of the experiment, were purchased from the Department of Laboratory Animal Science, Peking University Health Science Center. All the rats were housed in separate cages under the following standard conditions: temperature (18–24°C), humidity (55–65%), and dark cycle (12 h-light/12 h-dark cycle), with ad libitum access to food and water. All experimental protocols were approved by the animal care and use committee of Peking University (approved number: LA2021371).

### 2.2. Animal Model of Oligoasthenozoospermia

A rat model of iOAZS was developed by intragastric administration of ornidazole (ORN), as described previously [24]. In brief, adult male rats were intragastrically administered with ORN at a dose of 400 mg/kg body weight once per day from day 1 to day 14. The control rats received a 0.2% carboxymethylcellulose sodium (CMC-Na) solution (vehicle of ORN) throughout the experiment. Development of the iOAZS rat model was determined by assessment of the epididymal sperm motility and count as follows.

### 2.3. Xianlu Oral Solution Administration for Animals

Xianlu oral solution (XL, a gift from Changchun Leiyunshang Pharmaceutical Company) was kept at room temperature before use. The iOAZS model rats were intragastrically administered low-dose XL (1.5 ml/kg/d), middle-dose XL (3.0 ml/kg/d), high-dose XL (6.0 ml/kg/d), or equal amounts of normal saline (NS) once per day from day 15 to day 35. Meanwhile, the XL- and NS-treated iOAZS model rats were intragastrically administered with ornidazole (ORN, 400 mg/kg/d) once per day to maintain the pathological state of idiopathic oligoasthenozoospermia. The effects of XL treatment on iOAZS rats were also determined by assessment of the epididymal sperm motility and count.

### 2.4. Enzyme-Linked Immunosorbent Assay (ELISA)

Serum samples of rats were collected and kept at 4°C before use. ELISA kits (MEIMIAN, China) were used to quantify the levels of follicle-stimulating hormone (FSH, MM-70867R1), luteinizing hormone (LH, MM-0624R1), testosterone (T, MM-0577R1), and blood urea nitrogen (BUN, MM-20555R1), and ELISA kits (MEIBIAO, China) were used to quantify the levels of alanine aminotransferase (ALT, MB-6892B) and aspartate aminotransferase (AST, MB-6891B) according to the manufacturer's instructions.

### 2.5. Hematoxylin and Eosin (H&E) Staining

Under deep anesthesia, the testes tissues of rats were removed quickly, fixed in 4% neutral buffered formalin, dehydrated through an ethanol series, and cleared twice in 100% xylene. For embedding, the testes tissues were transferred to pure paraffin wax for 1 h at 60°C. For H&E staining, 5 *µ*m testicular sections were dewaxed in xylene, rehydrated through ethanol series, and then stained with H&E. Images were acquired using a light microscope (OLYMPUS, Tokyo, Japan).

### 2.6. Computer-Assisted Sperm Analysis (CASA)

Cauda epididymal sperm of rats were collected immediately after euthanasia and prepared as described in a previous article [25]. In brief, two caudal epididymides were placed in 2 ml preheated phosphate buffer saline (PBS), slightly cut into three pieces and incubated for 5 min at 37°C in a 5% CO_2_ incubator. Ten microliters of the sperm suspension were used for the assessment of sperm motility and concentrated by using a CASA system (WLJY-9000, Beijing Weili New Century Science and Technology Development Co., Ltd, Beijing, China). The following parameters were evaluated: rapid progressive motility (grade A sperm, %), progressive motility (grade A + B sperm, %), and sperm concentration (million/ml), as well as the parameters of sperm motility such as straight-line velocity (VSL, *μ*m/s), curvilinear velocity (VCL, *μ*m/s), average path velocity (VAP, *μ*m/s), amplitude of lateral head displacement (ALH, *μ*m), linearity (LIN, %), and straightness (STR, %) were also evaluated.

### 2.7. Sperm Morphological Staining

Sperm morphological staining was performed by the Diff–Quik method using the sperm morphological fast staining kit (G2572, Solarbio, Beijing, China) according to the manufacturer's instructions. In brief, 20 *μ*l of the sperm suspension were added to the slides, smeared, and dried in air. The slides were soaked in Diff staining buffer 1 for 20 s and then soaked in Diff staining buffer 2 for 10 s. Images were acquired using a light microscope (OLYMPUS, Tokyo, Japan).

### 2.8. Sperm DNA Staining

Sperm DNA staining was performed by the AO method using the sperm nucleus DNA staining kit (DA1210A, Leagene Biotechnology, Beijing, China) according to the manufacturer's instructions. In brief, 20 *μ*l of the sperm suspension was added to the slides, smeared, and dried in air. The slides were fixed in the stationary buffer for 15 min, washed in ddH_2_O, and swung to remove the redundant water. Then, the slides were stained in AO staining working solution for 5 min. The slides were observed under a confocal microscope (Zeiss LSM710), and images were captured with ZEISS ZEN software (Carl Zeiss).

### 2.9. Detection of Mitochondrial Membrane Potential (MMP)

For the MMP analysis of testicular tissues in rats, the mitochondrial membrane potential assay kit with JC-1 (C2006, Beyotime Biotechnology, Jiangsu, China) was used following the manufacturer's instructions.

### 2.10. Western Blotting

A piece of testicular tissue from the rats was immediately homogenized in ice-cold RIPA lysis buffer containing 1 mM phenylmethanesulfonyl fluoride (P0013B, Beyotime Biotechnology, Jiangsu, China). The homogenates were centrifuged at 12,000 *g* for 10 min at 4°C to yield the total protein extract in the supernatant. The concentration of protein was measured with a bicinchoninic acid (BCA) assay kit (Pierce/Thermo Scientific), and equal amounts of protein samples (60 *μ*g) were denatured and then separated in 10% sodium dodecyl sulfate-polyacrylamide gels and then transferred onto PVDF membranes. The membranes were incubated with the following primary antibodies overnight at 4°C: rabbit monoclonal anti-Bcl-2 (1 : 1000, Cell Signaling Technology (CST), cat^#^ 3498), rabbit polyclonal anti-caspase-3 (1 : 1000, CST cat^#^ 9662), rabbit polyclonal anti-4-hydroxynonenal (4-HNE) (1 : 1000, Abcam, cat^#^ ab48506), and mouse monoclonal anti-*α*-tubulin (1 : 1000, CST, cat^#^ 3873). The membranes were washed in TBST and incubated with the indicated horseradish peroxidase-conjugated secondary antibody including goat anti-rabbit IgG antibody (1 : 2000, Biodragon Immunotechnologies, Suzhou, Jiangsu, China, cat^#^ BF03008) and goat anti-mouse IgG antibody (1 : 2000, Biodragon Immunotechnologies, cat^#^ BF03001) for 1 h at room temperature and then washed in TBST. Immunoreactive bands were visualized by using a Tanon 5200 chemiluminescence detection system (Tanon, Shanghai, China). The bands were quantified with a computer-assisted imaging analysis system (ImageJ, NIH).

### 2.11. Oxidative Stress Assessments

Total antioxidant capacity (cat^#^ S0119, total antioxidant capacity assay kit with ABTS method), glutathione peroxidase (GPx) (cat^#^ S0058, total glutathione peroxidase assay kit with NADPH), and superoxide dismutase (SOD) (cat^#^ S0109, total superoxide dismutase assay kit with NBT) activities as well as the levels of hydrogen peroxide (cat^#^ S0038, hydrogen peroxide assay kit) and malondialdehyde (MDA) (cat^#^ S0131M, lipid peroxidation MDA assay kit) in testicular tissues of rats were measured using commercial kits purchased from Beyotime Biotechnology according to the manufacturer's instructions.

### 2.12. Statistical Analysis

All the statistical analyses were performed with GraphPad Prism 8.0.2 (GraphPad Software, La Jolla, CA, USA). The data were presented as the mean ± standard error of the mean (mean ± SEM). One-way ANOVA followed by Sidak's post hoc test was used for multiple comparisons of three groups. The significant differences between groups are represented as ^*∗*^*P* < 0.05, ^*∗∗*^*P* < 0.01, and ^*∗∗∗*^*P* < 0.001.

## 3. Results

### 3.1. Effects of XL Treatment on the Testis Index and Serum Hormone Levels of OAZS Rats

**Figure 1:**
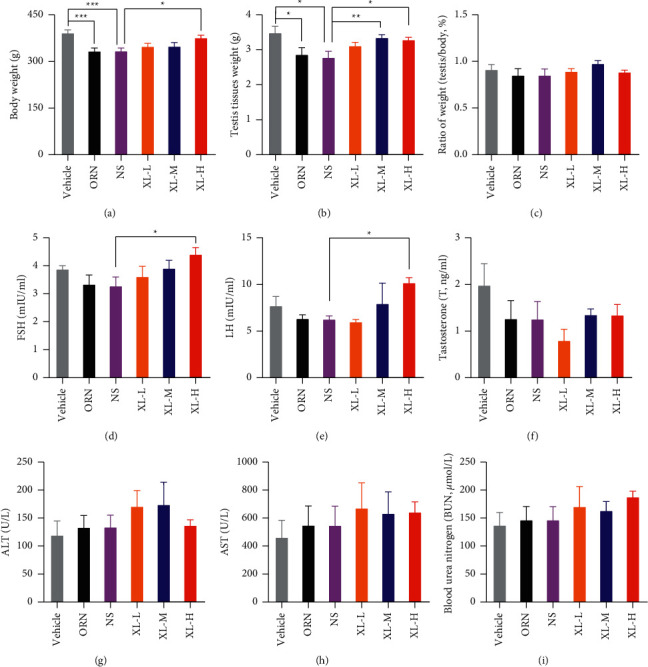
Improvement of FSH and LH levels in the serum of iOAZS rats with XL treatment. (a–c) Body weight, testis tissue weight, and the ratio of testis tissue weight to body weight. All data were presented as mean ± SEM. ^*∗*^*P* < 0.05, ^*∗∗*^*P* < 0.01, and ^*∗∗∗*^*P* < 0.001. One-way ANOVA followed by Sidak's post hoc test, *n* = 8 to 10 rats per group. (d–f) The levels of follicle-stimulating hormone (FSH), luteinizing hormone (LH), and testosterone (T). (g–i) The levels of aminotransferase (ALT), aspartate aminotransferase (AST), and blood urea nitrogen (BUN). All data were presented as mean ± SEM. ^*∗*^*P* < 0.05. One-way ANOVA followed by Sidak's post hoc test, *n* = 5 to 6 rats per group.

To evaluate the effects of XL treatment on iOAZS rats, we first examined the alterations in body and testicular tissue weights of the rats in different groups. The body and testis weights of iOAZS rats were decreased, although the testis index (ratio of testis/body weight) was not altered. Additionally, a high-dose XL (XL-H) treatment improved the body and testis weights of iOAZS rats (Figures 1(a)–1(c)). Then, using ELISA, we found an increase in the FSH and LH levels of high-dose XL-treated rats, but the testosterone level was not changed (Figures 1(d)–1(f)). Moreover, we found that ORN and XL treatment had no side effects on the liver and kidney since the levels of ALT, AST, and urea were not changed in ORN- and XL-treated rats (Figures 1(g)–1(i)). These results suggest that XL treatment could improve testis spermatogenesis in iOAZS rats.

### 3.2. XL Treatment Improved Testis Spermatogenesis in iOAZS Rats

**Figure 2:**
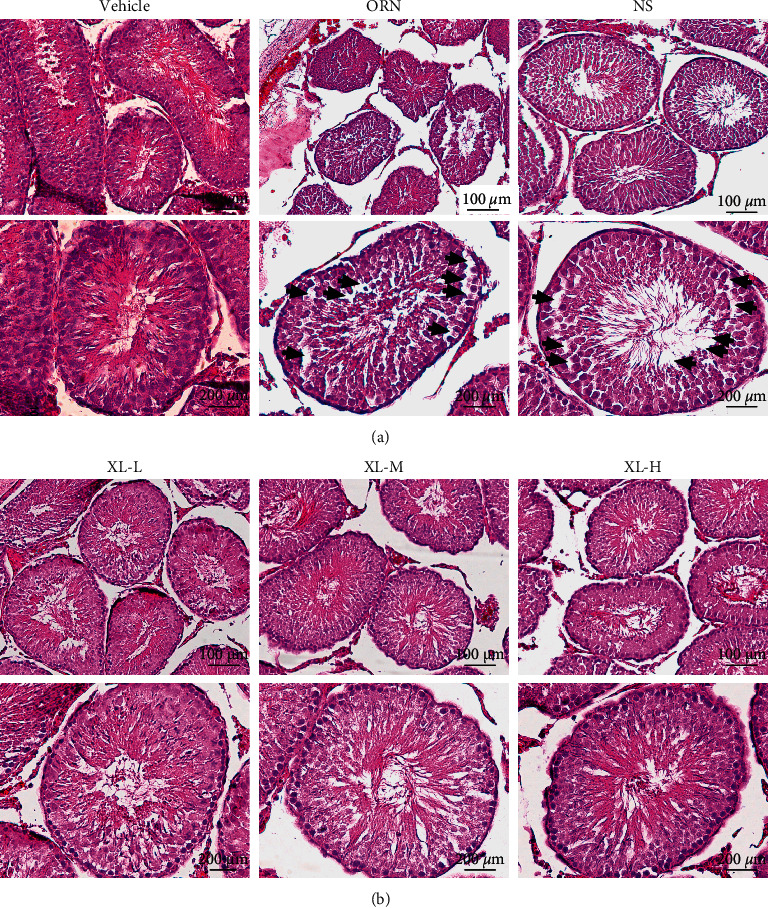
Ameliorated testis spermatogenesis of iOAZS rats with XL treatment. (a) H&E staining of rat testis tissues of CMC-Na (vehicle)-, ORN-, and normal saline (NS)-treated rats. (b) H&E staining of rat testis tissues of low-dose XL (XL-L), medium-dose XL (XL-M), and high-dose XL (XL-H) treated rats. Scale bar = 100 *μ*m or 200 *μ*m. Arrows indicate the areas without spermatogenic cells.

To further determine whether XL treatment had therapeutic effects on iOAZS rats, we first evaluated testis spermatogenesis in rats. The hematoxylin and eosin (H&E) staining of the testis tissue showed that, on day 35 after exposure of ORN to rats, the spermatogenic cells and spermatids in the seminiferous tubules were decreased and the seminiferous tubules were disordered in the testis tissues in iOAZS rats compared with vehicle-treated rats (Figure 2(a)). Moreover, we found that XL treatment improved testis spermatogenesis in iOAZS rats in a dose-dependent manner, and the disruption of seminiferous tubules and decrease of spermatogenic cells were alleviated after XL treatment in iOAZS rats (Figure 2(b)). These results suggest that XL treatment ameliorated spermatogenesis in iOAZS rats.

### 3.3. XL Treatment Enhanced the Sperm Concentration and Motility of iOAZS Rats

**Figure 3:**
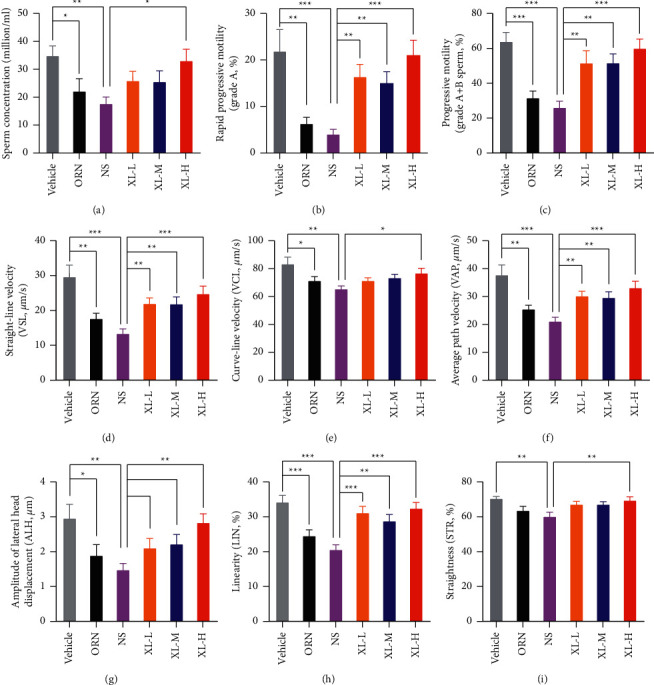
Enhanced sperm concentration and motility of iOAZS rats with XL treatment. (a–c) Sperm concentration, rapid progressive motility (grade A sperm), and progressive motility (grade A + B sperm). (d) Straight-line velocity (VSL), (e) curve-line velocity (VCL), (f) average path velocity (VAP), (g) amplitude of lateral head displacement (ALH), (h) linearity (LIN), and (i) straightness (STR). All data were presented as mean ± SEM. ^*∗*^*P* < 0.05, ^*∗∗*^*P* < 0.01, and ^*∗∗∗*^*P* < 0.001. One-way ANOVA followed by Sidak's post hoc test, *n* = 10 to 11 rats per group.

To further enhance our understanding of how XL treatment improved spermatogenesis in iOAZS rats, we examined the alteration of sperm quality in ORN- and XL-treated rats. Using the CASA technique, we found a significant reduction in sperm concentration and sperm motility, including grade A and grade A + B sperm in iOAZS rats compared with vehicle controls (Figures 3(a)–3(c)). Other parameters of sperm motility, including straight-line velocity (VSL), curve-line velocity (VCL), average path velocity (VAP), amplitude of lateral head displacement (ALH), linearity (LIN), and straightness (STR), were consistently decreased in iOAZS rats (Figures 3(d)–3(i)). Additionally, a high-dose XL treatment significantly improved the sperm quality of iOAZS rats, as indicated by augmented sperm concentration and sperm motility including grade A sperm, grade A + B sperm and other parameters of sperm motility such as VSL, VCL, VAP, ALH, LIN, and STR (Figures 3(a)–3(i)). Consistently, low- and medium-dose XL treatment enhanced sperm motility (grade A and grade A + B sperm) and some parameters of sperm motility such as VSL, VAP, and LIN (Figures 3(a)–3(i)). However, abnormal sperm morphology and DNA fragment index (single-stranded DNA/double-stranded DNA) were not altered by ORN or XL treatment in rats (Supplementary Figure 1). These data suggest that XL treatment enhanced the sperm concentration and motility of iOAZS rats.

### 3.4. Effects of XL Treatment on the Mitochondrial Membrane Potential (MMP) and Apoptosis Status of iOAZS Rats

**Figure 4:**
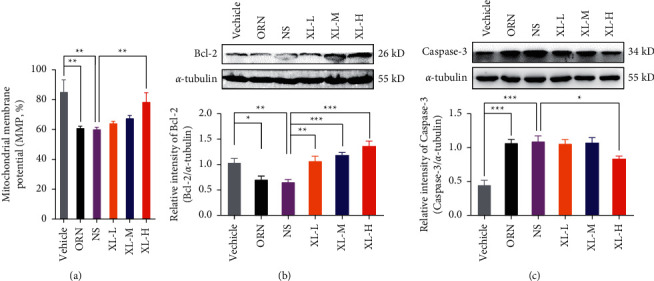
Improved mitochondrial membrane potential (MMP) and apoptosis status of iOAZS rats with XL treatment. (a) The mitochondrial membrane potential of testis tissues. (b, c) Expression of Bcl-2 and caspase-3 protein in the testis tissues. All data were presented as mean ± SEM. ^*∗*^*P* < 0.05, ^*∗∗*^*P* < 0.01, and ^*∗∗∗*^*P* < 0.001. One-way ANOVA followed by Sidak's post hoc test, *n* = 4 to 5 rats per group.

To further clarify the underlying mechanism contributing to the improvement of XL treatment in iOAZS rats, we first evaluated the mitochondrial membrane potential (MMP) and apoptosis status of ORN- and XL-treated rats. Using a JC-1 assay kit, we found that MMP was reduced in the testis tissues of OAZS rats and was increased after high-dose XL treatment of iOAZS rats (Figure 4(a)). Western blotting results showed that XL treatment abrogated the reduced abundance of Bcl-2 protein in the testis tissues of OAZS rats (Figure 4(b)) and only high-dose XL treatment attenuated the increased level of caspase-3 protein in the testis tissues of iOAZS rats (Figure 4(c)). These data indicate that XL treatment ameliorated the mitochondrial membrane potential (MMP) and apoptosis status in the testis tissues of iOAZS rats.

### 3.5. Effects of XL Treatment on the Oxidative Stress Status of iOAZS Rats

**Figure 5:**
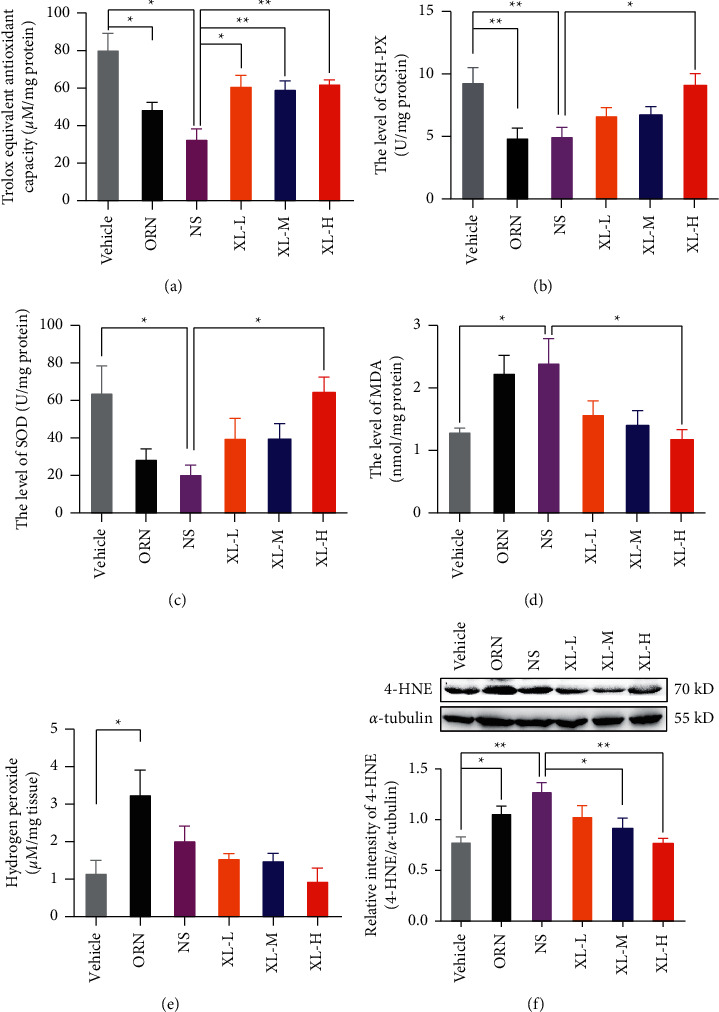
Alleviated oxidative stress status in the testis tissue of iOAZS rats with XL treatment. (a–c) Total antioxidant capacity represented by trolox-equivalent antioxidant capacity, total glutathione peroxidase (GPx), and total superoxide dismutase (SOD). (d–f) The level of hydrogen peroxide, lipid peroxidation represented by malondialdehyde (MDA), and protein expression of 4-hydroxynonenal (4-HNE). All data were presented as mean ± SEM. ^*∗*^*P* < 0.05 and ^*∗∗*^*P* < 0.01. One-way ANOVA followed by Sidak's post hoc test, *n* = 4 to 8 rats per group.

We also found a decline in total antioxidant capacity, total GPx, and SOD activities, as well as a rise in the level of hydrogen peroxide, MDA, and protein expression of 4-hydroxynonenal (4-HNE) in the testis tissues of iOAZS rats (Figures 5(a)–5(f)). Moreover, high-dose XL treatment ameliorated the augmented oxidative stress in the testis tissues of iOAZS rats, as manifested by enhanced total antioxidant capacity, total GPx, and SOD activities and attenuation of the level of MDA and protein expression of 4-HNE (Figures 5(a)–5(f)). Taken together, we suggest that XL treatment exerts its therapeutic actions on iOAZS rats by ameliorating the mitochondrial membrane potential, apoptosis status, and oxidative stress status in testis tissues.

## 4. Discussion

In this study, we demonstrated that Xianlu oral solution treatment has a therapeutic effect on iOAZS by ameliorating the serum hormone levels, mitochondrial membrane potential, apoptosis status, and oxidative stress status in the testis tissues. This study provides a novel mechanism for Xianlu oral solution treatment on iOAZS, and Xianlu oral solution may be used as a traditional Chinese medicine (TCM) therapy for male infertility caused by iOAZS in clinical practice.

Multiple causes can lead to oligoasthenozoospermia (OAZS), including infections and varicocele-induced reproductive system disease, sex chromosome abnormalities (Klinefelter's syndrome and Y chromosome microdeletions), and defects in spermatozoa flagella microstructure or function such as primary ciliary dyskinesia (PCD) or multiple morphological abnormalities of the sperm flagellum (MMAF) [26, 27]. Prostatitis or varicocele could result in augmentation of the autoimmune response and seminal inflammatory factors, increased apoptosis, increased oxidative stress, and spermatozoa DNA damage, resulting in oligoasthenoteratozoospermia [28–31]. However, there were also some cases without clear causes, categorized as idiopathic oligoasthenozoospermia (iOAZS). To date, there have been no ideal therapies for iOAZS, although methods such as treating infection or varicocele by antibiotics or surgery, changing in lifestyle, avoiding toxic environmental exposures, and maintaining regular intercourse and ejaculation have been established for OAZS, as well as the application of intracytoplasmic sperm injection (ICSI) for some severe OAZS patients caused by genetic factors [14–17].

Traditional Chinese medicine (TCM) has been widely used for the treatment of male infertility. Wenshen Shengjing decoction treatment could repair testicular damage and increase testosterone levels in the testes of cyclosporine-induced OAZS mice [20]. The components of Xianlu oral solution (XL), such as *Cuscuta chinensis* Lam., *Lycium barbarum* L., Fructus Ligustri Lucidi, and *Panax ginseng* C. A. Meyer, could ameliorate spermatogenic dysfunction and improve the sperm quality of different model animals by attenuating testis oxidative damage and apoptosis of spermatogenic cells, as well as by augmenting sex hormones [23, 32–35]. Our results showed that high-dose Xianlu oral solution (XL) treatment improved the FSH and LH but not the testosterone level in the serum, as well as without side effects on liver and kidney function of ORN-induced iOAZS rats. We think XL may influence the serum hormone levels of OAZS rats through the hypothalamic-pituitary-gonad axis (HPG). Furthermore, we found that XL treatment also ameliorated testis spermatogenesis in iOAZS rats by alleviating the disruption of seminiferous tubules and increasing the spermatogenic cells in a dose-dependent manner. Likewise, Sheng-Jing-San treatment improved the sperm motility of AZS model rats, and Wuzi Yanzong (WZYZ) formula administration restored the destroyed testicular structure of oligoasthenozoospermic model rats [18, 19]. *Cistanche tubulosa* (CT), echinacoside (ECH), and phenylethanol glycosides from *C. tubulosa* (CPhGs) treatment also attenuated the poor sperm quality and testicular toxicity of bisphenol A- or hydrocortisone-treated animals [20, 21]. In line with these findings, we found that high-dose XL treatment improved the sperm quality of ORN-induced iOAZS rats, including the augmentation of sperm concentration and sperm motility, including grade A sperm, grade A + B sperm, and other parameters of sperm motility such as VSL, VCL, VAP, ALH, LIN, and STR. Consistently, low- and medium-dose XL treatment enhanced sperm motility (grade A and grade A + B sperm) and some parameters of sperm motility, such as VSL, VAP, and LIN. We also evaluated the abnormal sperm morphology and DNA fragment index of XL-treated iOAZS rats since DNA fragmentation and epigenetic abnormalities of sperm were associated with sperm quality of men with infertility [36–39]; however, our results showed that XL treatment had no effect on abnormal sperm morphology and DNA fragment index of iOAZS rats. We speculated that these results may be due to the lack of alterations in abnormal sperm morphology and DNA fragment index after ORN treatment.

During the occurrence of spermatogenesis disorder, abnormal mitochondrial function, cell apoptosis, and oxidative stress are also involved. Excessive ROS generation of mitochondria, abnormal assembly of mitochondria, or structural defects in mitochondrial membranes are associated with asthenozoospermia or oligozoospermia [40–44]. Consistently, we demonstrated that mitochondrial membrane potential (MMP) was decreased in the testis tissues of iOAZS rats and was increased after high-dose XL treatment of iOAZS rats. We also showed that XL treatment mitigated the cell apoptosis of iOAZS rats since XL treatment abrogated the reduced abundance of Bcl-2 protein and attenuated the increased level of caspase-3 protein in the testis tissues of iOAZS rats. Likewise, the new Wenshen Shengjing decoction, *Cuscuta chinensis* Lam. and *Lycium barbarum* L. treatment reduced sperm cell apoptosis by regulating the expression of Bcl-2, BAD, and BAX, thus improving sperm counts of *Tripterygium wilfordii* Hook. polyglycoside-treated rats [31, 43].

Oxidative stress occurs when there is an imbalance between ROS and antioxidants, and oxidative stress in testis tissues can be caused by varicocele and infection, abuse of alcohol and drugs, radiation, metabolic diseases, and mental stress [15, 45, 46]. Male infertility is associated with excessive oxidative stress and lipid peroxidation of sperm [38, 39, 47]. It is well known that oxidative stress is a major cause of male infertility since it can induce sperm nuclear and mitochondrial DNA (mtDNA) damage, telomere shortening, epigenetic alterations, and Y chromosomal microdeletions [48]. Spermatozoa are more susceptible to oxidative stress and lipid peroxidation because plasma membrane of spermatozoa contains a large amount of polyunsaturated fatty acids (PUFAs), and oxidative stress and lipid peroxidation will lead to the generation of MDA and 4-HNE, causing damage to spermatozoa [49, 50]. Y chromosomal microdeletions caused by oxidative stress during the differentiation and maturation processes in the male reproductive tract may lead to male infertility, such as azoospermia or severe oligozoospermia [51]. Epigenetic abnormalities such as hypomethylation induced by oxidative stress were also found in oligozoospermic men [36, 37]. We also found a decline in total antioxidant capacity, total GPx, and SOD activities, as well as a rise in the level of hydrogen peroxide, MDA, and protein expression of 4-HNE in the testis tissues of iOAZS rats. Moreover, the high-dose XL treatment ameliorated the augmented oxidative stress in the testis tissues of iOAZS rats, such as the enhanced total antioxidant capacity, total GPx, and SOD activities and attenuation of the level of MDA and protein expression of 4-HNE. Likewise, *Epimedium brevicornu* Maxim (Yinyanghuo) and Fructus Ligustri Lucidi (Nvzhenzi) may have some therapeutic effects on OAZS by alleviating hydrogen peroxide-induced oxidative damage [52, 53].

## 5. Conclusion

In conclusion, this study demonstrates that intragastric administration of ORN to rats could produce a reduction of sperm concentration and motility, whereas Xianlu oral solution treatment increased the sperm concentration and motility by increasing the serum FSH and LH hormone levels, augmenting the mitochondrial membrane potential, mitigating apoptosis, and ameliorating oxidative stress status in the testis tissues of ORN-treated rats. Xianlu oral solution may be used as a therapy for iOAZS patients in clinical practice.

## Data Availability

The data that support the findings in this study are available from the corresponding author upon reasonable request.

## Supplementary Materials

Supplementary Materials
*Supplementary Figure 1*. No alterations in sperm morphology or DNA integrity were observed in iOAZS rats treated with XL. (A) Representative images and summary of sperm morphology. The red arrow indicates the sperm with abnormal morphology. All data were presented as mean ± SEM. One-way ANOVA followed by Sidak's post hoc test, *n* = 24 visual fields from 6 rats per group. (B) Representative images of sperm DNA integrity. Green indicates the double-stranded DNA, and red indicates the single-stranded DNA of sperm.
