# Supplementary figures and images for: Therapeutic Effects of Xianlu Oral Solution on Rats with Oligoasthenozoospermia through Alleviating Apoptosis and Oxidative Stress
Source: Evid Based Complement Alternat Med. 2022 Jun 18;2022:1269530. doi: 10.1155/2022/1269530 (PMC9233599; doi:10.1155/2022/1269530)

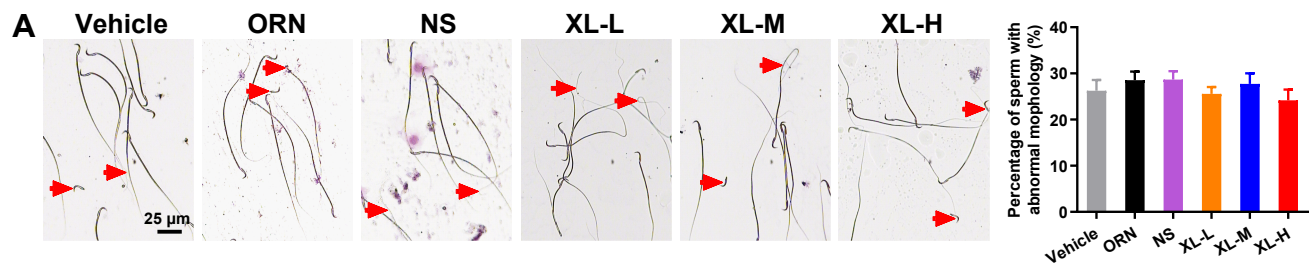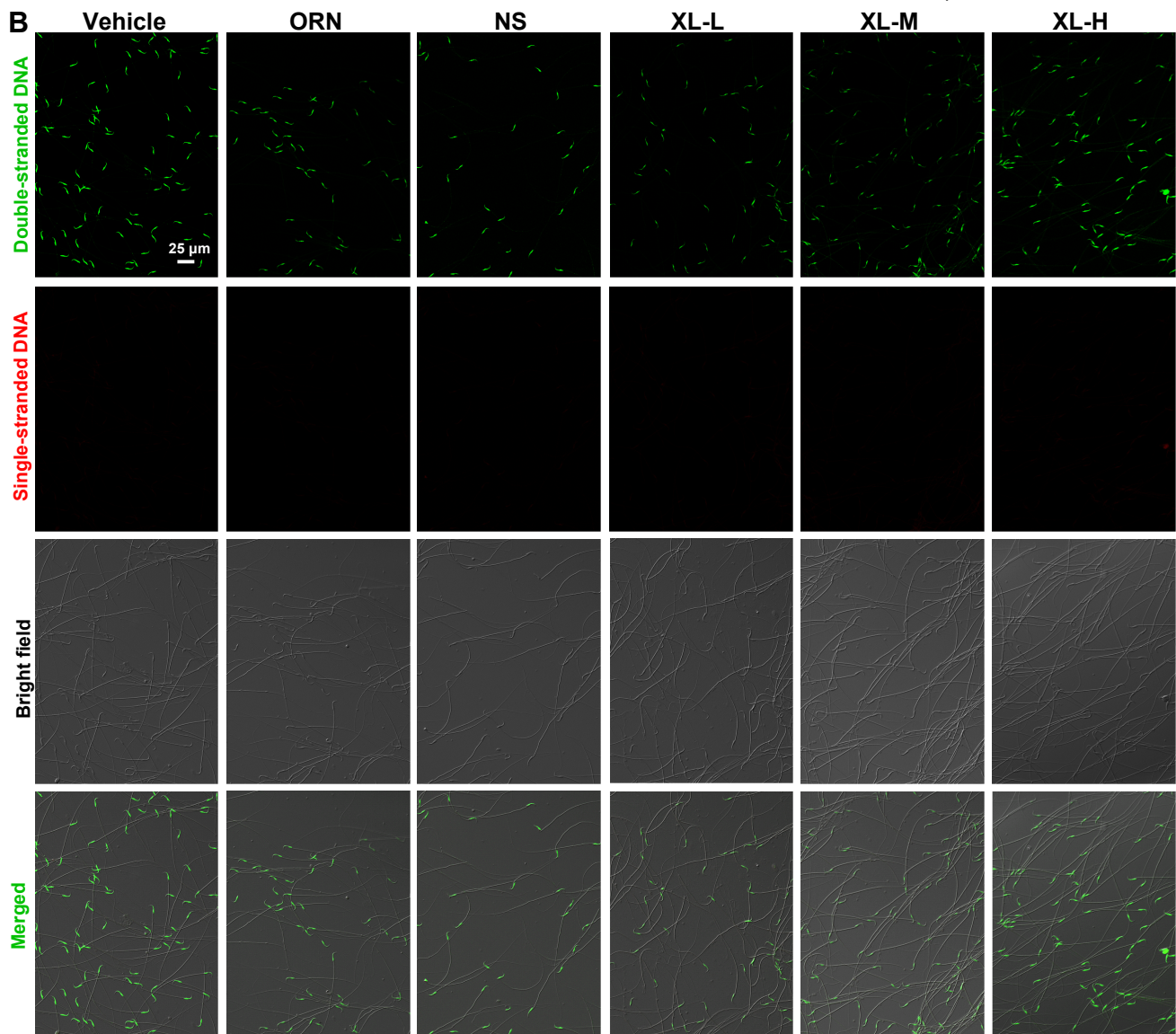

Supplement: Supplementary Materials — Supplementary Figure 1. No alterations in sperm morphology or DNA integrity were observed in iOAZS rats treated with XL. (A) Representative images and summary of sperm morphology. The red arrow indicates the sperm with abnormal morphology. All data were presented as mean ± SEM. One-way ANOVA followed by Sidak's post hoc test, n = 24 visual fields from 6 rats per group. (B) Representative images of sperm DNA integrity. Green indicates the double-stranded DNA, and red indicates the single-stranded DNA of sperm. [file 1269530.f1.pdf]
